# Association between sleep duration and depression in menopausal women: a population-based study

**DOI:** 10.3389/fendo.2024.1301775

**Published:** 2024-02-19

**Authors:** Feng Zhang, Long Cheng

**Affiliations:** ^1^ Department of Medicine, Shandong Liming Science and Technology Vocational College, Jinan, China; ^2^ Department of Anesthesiology, Beijing Jishuitan Hospital, Capital Medical University, Beijing, China

**Keywords:** Patient Health Questionnaire-9, sleep duration, menopausal women, National Health and Nutrition Examination Survey (NHANES), women

## Abstract

**Aims:**

This research investigated menopausal women older than 50 years to find whether there were any independent relationships between the duration of sleep they got and their prevalence of depression.

**Methods:**

National Health and Nutrition Examination Survey (NHANES) datasets from 2011-2020 were utilized in a cross-sectional study. Using multivariate linear regression models, the linear relationship between sleep duration and depression in menopausal women was investigated. Fitted smoothing curves and thresholds impact evaluation were used to investigate the nonlinear relationship. Then, subgroup analyses were performed according to smoking, drinking alcohol, diabetes, hypertension, heart disease, and moderate activities.

**Results:**

This population-based study included a total of 3,897 menopausal women (mean age 65.47 ± 9.06 years) aged≥50 years; 3,159 had a depression score <10, and 738 had a depression score≥10. After controlling for all covariates, the prevalence of depression was 17% higher among participants with short sleep duration [*OR*=1.17, 95%CI=(0.65, 1.70), *P*<0.0001] and 86% [*OR*=1.86, 95%CI=(1.05, 2.66), *P*<0.0001] compared to participants with normal sleep duration. In subgroup analyses stratified by smoking and diabetes, the sleep duration and depression scores of non-smokers [*β*=-0.18, 95%CI= (-0.33, -0.02), *P*=0.0241] and diabetics were independently negatively correlated [*β*=-0.32, 95%CI= (-0.63, -0.01), *P*=0.0416]. Using a two-segment linear regression model, we discovered a U-shaped relationship between sleep duration and depression scores with an inflection point of 7.5 hours. Less than 7.5 hours of sleep was associated with an increased risk of developing depression [*β*=-0.81, 95%CI= (-1.05, -0.57), *P*<0.001]. However, sleeping more than 7.5 hours per night increased the risk of depression considerably [*β*=0.80, 95%CI= (0.51, 1.08), *P*<0.001].

**Conclusions:**

Depression is associated with sleep duration in menopausal women. Insufficient or excessive sleep may increase the risk of depression in menopausal women.

## 1 Background

Menopause is considered a significant turning point in a woman’s life cycle due to the failure of ovarian function, resulting in a decline in estrogen and progesterone levels, which has a significant impact on a woman’s physical and mental health (1, 2). By 2030, it is predicted that 1,2 billion women worldwide will have reached menopause (3). There is evidence that estrogen deficiency increases the risk of depression during menopause (4). In addition, depression may increase the risk of physical health issues such as cardiovascular disease, diabetes mellitus, metabolic syndrome, osteoporosis, and bone fractures and have a negative impact on the quality of life, social life, and professional life of menopausal women (5). As a consequence, menopausal depression has been documented as a significant global mental health issue for women.

Previous research has demonstrated a significant association between depression and poor sleep quality (6). Good sleep is essential for restoring and maintaining bodily functions (7). Approximately 28%-63% of women report sleep problems during menopause (8). Menopausal symptoms and hormone levels influence poor sleep and are one of the most prevalent health issues reported by menopausal women throughout the reproductive cycle (9). Generally, individuals’ recommended minimum and maximum daily sleep duration are seven and nine hours, respectively (10). Notably, approximately 35 percent of menopausal women sleep less than six hours per night (11). Furthermore, the shorter the nighttime sleep duration of menopausal women, the more severe their symptoms (12). Moreover, unfavorable sleep practices can impact the subjective well-being of menopausal women (7).

Poor sleeping habits are known to have several unhealthy effects. Epidemiological and meta-analytic evidence supports a causal association between short and extended sleep duration and adverse health outcomes (13–15). Many studies to date have investigated the function of sleep duration in the development of depression. A prospective study revealed that both short and long sleep durations are substantially associated with an increased risk of depression in adults (16). Other studies support the idea that not enough or too much sleep increases the risk of developing depression (17, 18). However, the relationship between sleep duration and depression in menopausal women is unclear.

Therefore, in the present study, we utilized a large sample of a population of menopausal women from the National Health and Nutrition Examination Survey (NHANES) to investigate the relationship between sleep duration (both sleep deprivation and excess) and depression in menopausal women.

## 2 Methods

### 2.1 Study population

**Figure 1:**
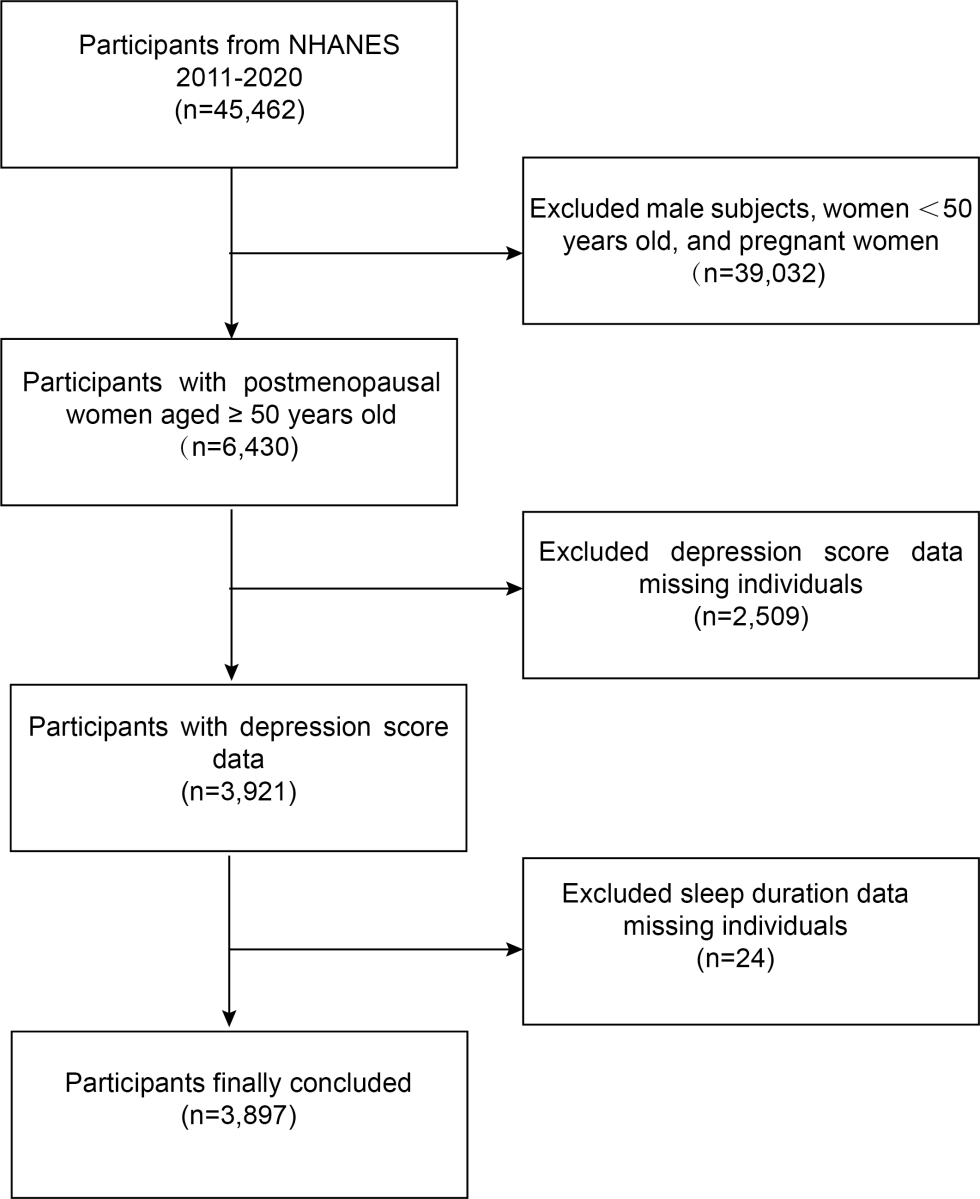
Flowchart for selecting participants. NHANES, National Health and Nutrition Examination Survey.

The NHANES is a representative study of the U.S. population that uses a complicated, multistage, and probabilistic sampling process that provides a wealth of data about the general health and nutrition of the U.S. population (19). The U.S. NHANES dataset for cycles 2011-2020 was used for this survey. The following was the inclusion standard: (i) women over 50 who are menopausal. The following were the exclusion standards: (i) participants who were pregnant (20) and (ii) participants whose sleep duration and depression scores were missing. 3,897 subjects in total were included in the study. The flowchart for an example selection is shown in **Figure 1**.

### 2.2 Menopausal status definitions

Based on the self-reported reproductive health questionnaire, menopausal status was determined. If a woman replied “no” to the question “Have you had at least one menstrual period in the past 12 months?” and then said “hysterectomy” or “menopause/change of life” in response to the following question, she was considered to be postmenopausal. On the NHANES website, you may get more information about the self-reported reproductive health questionnaire (CDC. questionnaire instruments (2022). Available at: https://wwwn.cdc.gov/nchs/nhanes/ContinuousNhanes/Questionnaires.aspx?BeginYear=2017.).

### 2.3 Assessment of exposure: sleep duration

Participants self-reported how much sleep they had on their typical workday or weekday. In 2011–2014, the NHANES participants’ daily sleep duration was obtained by asking them the following question: “How many (hours) of sleep did you get?” The definition of sleep duration for the 2015–2020 cycle was based on the inquiry, “How much sleep do you typically get at night on weekdays or workdays?” Recorded times were categorized as short (less than 7 hours per night), normal (7-9 hours per night), and long (more than 9 hours per night) (21).

### 2.4 Assessment of outcome: depression

The Patient Health Questionnaire (PHQ-9), a nine-item screening tool that inquires about the frequency of depression symptoms in the previous two weeks, was used to assess depressed symptoms (22). The nine answer choices, which range from “not at all,” “several days,” “more than half the days,” and “nearly every day,” are based on the PHQ-9 and correspond to scores of 0 to 3, respectively. The PHQ-9 is a reliable and valid diagnostic tool grounded in the DSM-V criteria, with a maximum possible total score of 27. A cut-off point at or exceeding ten has demonstrated a sensitivity of 88% for detecting major depression and an equivalent specificity of 88% (23). Therefore, we separated the PHQ-9 scores of the subjects into two categories:<10 (no depression) and≥10 (depression) (22).

### 2.5 Assessment of covariates

The choice of covariates is guided by established research findings in the extant literature and rational arguments. Covariates included age, race, education level, smoking, drinking alcohol, diabetes, hypertension, heart disease (13), moderate activities, family income to poverty ratio, body mass index (BMI) (24), alanine transaminase (ALT), aspartate aminotransferase (AST), high-density lipoprotein cholesterol (HDL-C), low-density lipoprotein cholesterol (LDL-C), triglyceride, and total cholesterol (25). Consult the NHANES Survey Methods and Analysis Guide for more details on variable collection approaches (https://wwwn.cdc.gov/nchs/nhanes/AnalyticGuidelines).

### 2.6 Statistical analysis

We relied on R (http://www.r-project.org) and EmpowerStats (http://www.empowerstats.com) for all statistical analyses. Subgroups with depression scores<10 and depression scores≥10 were used in the baseline tables for the study population, and continuous variables were statistically defined by mean values plus or minus standard deviation (SD) and weighted linear regression models. Multivariate linear regression analysis determined the beta values and 95% confidence intervals between the sleep duration and depression scores. Three models were used to construct the multivariate test: model 1, with no variable adjusted; model 2, with age and race changed; and model 3, with all covariates corrected. Simultaneous smoothed curve fits were performed by adjusting the same variables from model 3. The link between sleep duration and depression scores and their inflection point was investigated using a threshold effects analysis model. *P*<0.05 was considered to be statistically significant.

## 3 Results

### 3.1 Baseline characteristics

The baseline characteristics of the individuals are displayed in **Table 1**. A total of 3,897 menopausal women with a mean age of 65.47 ± 9.06 years were included based on the inclusion and exclusion criteria. Higher PHQ-9 score subjects were more likely to be non-Hispanic white, higher education, non-smokers, former alcohol users, non-diabetic, hypertension, non-heart disease, have a lower yearly household income, have a high BMI, high ALT, high AST, low HDL-C, and high triglycerides. Additionally, PHQ-9 scores were more significant in patients with short sleep duration.

**Table 1:** Characteristics of the participants.

Characteristics	PHQ < 10 (n = 3159)	PHQ ≥ 10 (n = 738)	*P*-value
Age (years)	65.88 ± 9.09	63.72 ± 8.81	<0.001
Race/ethnicity, n (%)			<0.001
Mexican American	296 (9.37)	101(13.69)	
Non-Hispanic White	1417(44.86)	278(37.67)	
Non-Hispanic Black	727(23.01)	168(22.76)	
Other races	719(22.76)	191(25.88)	
Education level, n (%)			<0.001
< high school	673(21.30)	250(33.88)	
High school	796(25.20)	186(25.20)	
> high school	1690(53.50)	302(40.92)	
Smoking, n (%)			<0.001
No	2750(87.05)	569(77.10)	
Yes	409(12.95)	169(22.90)	
Drinking alcohol, n (%)			<0.001
Never	1426(45.14)	390(52.85)	
Ever	1733(54.86)	348(47.15)	
Diabetes, (%)			<0.001
No	2497(79.04)	496(67.21)	
Yes	662(20.96)	242(32.79)	
Hypertension, n (%)			<0.001
No	1255(39.73)	219(29.67)	
Yes	1904(60.27)	519(70.33)	
Heart disease, n (%)			<0.001
No	2999(94.94)	666(90.24)	
Yes	160(5.06)	72(9.76)	
Moderate activities, n (%)			0.214
No	2160(68.38)	522(70.73)	
Yes	999(31.62)	216(29.27)	
Sleep duration, n (%)			<0.001
<7	907(28.71)	289(39.16)	
7-9	1955(61.89)	338(45.80)	
>9	297(9.40)	111(15.04)	
Income to poverty ratio	2.54 ± 1.59	1.74 ± 1.33	<0.001
BMI (kg/m^2^)	30.43 ± 7.71	32.65 ± 8.69	<0.001
ALT (IU/L)	20.82 ± 12.61	22.56 ± 15.24	0.002
AST (IU/L)	23.54 ± 10.61	25.23 ± 19.06	0.002
HDL-C (md/dL)	59.59 ± 17.19	56.58 ± 17.45	<0.001
LDL-C (md/dL)	116.41 ± 37.98	118.03 ± 42.70	0.489
Triglyceride (md/dL)	117.89 ± 65.88	135.71 ± 83.10	<0.001
Total cholesterol (md/dL)	200.71 ± 42.18	201.77 ± 45.74	0.560

Mean ± SD for continuous variables: *P* value was calculated by weighted linear regression model. % for categorical variables: *P* value was calculated by weighted chi-square test.

BMI, body mass index; ALT, alanine transaminase; AST, aspartate aminotransferase; HDL-C, high-Density Lipoprotein; LDL-C, low-Density Lipoprotein Cholesterol.

### 3.2 Associations between sleep duration and depression

**Figure 2:**
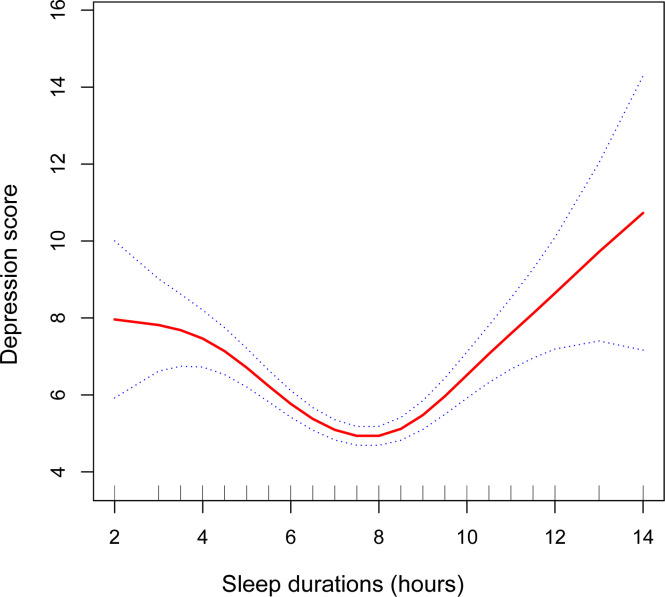
The association between sleep duration and depression. The smooth curve fit between variables is shown by the solid red line. The 95% confidence interval from the fit is shown by blue bars. A number of variables were adjusted, including age, race, educational level, smoking, drinking alcohol, diabetes, hypertension, heart disease, moderate activities, family income-to-poverty ratio, BMI, ALT, AST, HDL-C, LDL-C, triglyceride, and total cholesterol.

The analysis results using multiple linear regression are shown in **Table 2**. In the unadjusted model [*β*=-0.18, 95%CI=(-0.28, -0.09), *P*=0.0002], sleep duration was negatively associated with depression scores. However, after adjusting for age and race variables, this significant negative correlation became nonsignificant in Model 2 [*β*=-0.15, 95%CI=(-0.24, -0.05), *P*=0.0026]. After adjusting for all covariates, sleep duration was not significantly correlated with depression scores in Model 3 (*P*>0.05). After controlling for all covariates, the prevalence of depression was 17% higher among participants with short sleep duration [*OR*=1.17, 95%CI=(0.65, 1.70), *P*<0.0001] and 86% higher among participants with long sleep duration [*OR*=1.86, 95%CI=(1.05, 2.66), *P*<0.0001], compared to participants with normal sleep duration. Further validating the nonlinear negative association between sleep duration and depression scores were the findings from smooth curve fitting (**Figure 2**).

**Table 2:** Association between sleep duration and depression in menopausal women.

Characteristics	Model 1 [*β* (95% CI)]	*P*-value	Model 2 [*β* (95% CI)]	*P*-value	Model 3 [*β* (95% CI)]	*P*-value
Sleep duration	-0.18 (-0.28, -0.09)	0.0002	-0.15 (-0.24, -0.05)	0.0026	-0.09 (-0.24,0.05)	0.1930
Hours of Sleep						
<7	1.56 (1.21, 1.92)	<0.0001	1.45 (1.09, 1.81)	<0.0001	1.17 (0.65, 1.70)	<0.0001
7-9	Reference		Reference		Reference	
>9	2.07 (1.54, 2.61)	<0.0001	2.10 (1.57, 2.63)	<0.0001	1.86 (1.05, 2.66)	<0.0001
*Stratified by* Smoking						
No	-0.17 (-0.27, -0.07)	0.0010	-0.15 (-0.25, -0.05)	0.0038	-0.18(-0.33, -0.02)	0.0241
Yes	-0.11 (-0.36, 0.15)	0.4176	-0.08 (-0.33, 0.18)	0.5494	0.17 (-0.22, 0.56)	0.3941
*Stratified by* Drinking alcohol						
Never	-0.21 (-0.35, -0.07)	0.0026	-0.19 (-0.33, -0.05)	0.0077	-0.13 (-0.34, 0.07)	0.1990
Ever	-0.16 (-0.29, -0.03)	0.0166	-0.10 (-0.23, 0.03)	0.1242	-0.02 (-0.21, 0.18)	0.8569
*Stratified by* Diabetes						
No	-0.15 (-0.26, -0.04)	0.0055	-0.10 (-0.21, 0.00)	0.0593	0.01 (-0.15, 0.17)	0.8774
Yes	-0.26 (-0.46, -0.06)	0.0126	-0.25 (-0.45, -0.05)	0.0152	-0.32(-0.63, -0.01)	0.0416
*Stratified by* Hypertension						
No	-0.10 (-0.25, 0.05)	0.1838	-0.07 (-0.22, 0.08)	0.3821	-0.00 (-0.24, 0.24)	0.9978
Yes	-0.23 (-0.35, -0.11)	0.0002	-0.19 (-0.32, -0.07)	0.0017	-0.16 (-0.33, 0.02)	0.0833
*Stratified by* Heart disease						
No	-0.17 (-0.26, -0.07)	0.0007	-0.12 (-0.22, -0.03)	0.0135	-0.10 (-0.25, 0.04)	0.1624
Yes	-0.22 (-0.68, 0.23)	0.3419	-0.21 (-0.67, 0.24)	0.3564	-0.04 (-0.72, 0.65)	0.9114
*Stratified by* Moderate activities						
No	-0.16 (-0.28, -0.05)	0.0051	-0.13 (-0.25, -0.01)	0.0283	-0.03 (-0.21, 0.14)	0.7088
Yes	-0.23 (-0.40, -0.06)	0.0068	-0.20 (-0.37, -0.03)	0.0203	-0.22 (-0.47, 0.04)	0.0971

Model 1: no covariates were adjusted. Model 2: Age and race were adjusted. Model 3: Age, race, educational level, smoking, drinking alcohol, diabetes, hypertension, heart disease, moderate activities, family income-to-poverty ratio, BMI, ALT, AST, HDL-C, LDL-C, triglyceride, and total cholesterol were adjusted.

In the subgroup analysis stratified by smoking, drinking alcohol, diabetes, hypertension, heart disease, moderate activities, the model is not adjusted for smoking, drinking alcohol, diabetes, hypertension, heart disease, moderate activities, respectively.

In subgroup analyses stratified by smoking, our results indicate that the negative association between hours of sleep and scores for depression is independently significantly negative in non-smokers [*β*=-0.18, 95%CI= (-0.33, -0.02), *P*=0.0241], but not statistically significant in any model for smokers (*P*>0.05). In addition, after adjusting for all variables, subgroup analyses stratified by diabetes revealed a negative association between length of sleep and depression scores among those with diabetes [*β*=-0.32, 95%CI= (-0.63, -0.01), *P*=0.0416]. However, after controlling for all variables, this negative correlation dissipated among non-diabetics (*P*>0.05). Adjusting for all variables eliminated the negative association between sleep duration and depression scores in subgroup analyses by drinking alcohol, hypertension, heart disease, and moderate activities (*P*>0.05).

### 3.3 Non-linearity and threshold effect analysis between sleep duration and depression

The smoothed curve fit showed the u-shaped relationship between sleep duration and depression scores with an inflection point of 7.5 hours after adjusting for all covariates (**Figure 2**). Sleeping for less than 7.5 hours was connected adversely with a risk of developing depression [*β*=-0.81, 95%CI= (-1.05, -0.57), *P*<0.001]. However, sleeping more than 7.5 hours per night significantly raised the risk of depression [*β*=0.80, 95%CI= (0.51, 1.08), *P*<0.001] (**Table 3**).

**Table 3:** Threshold effect analysis of sleep duration on incident depression.

Sleep duration	Adjusted *β*(95%CI)	*P*-value
Fitting by the standard linear model	-0.09 (-0.24, 0.05)	0.1933
Fitting by the two-piecewise linear model		
Inflection point	7.5	
Sleep duration < 7.5 hours	-0.81(-1.05, -0.57)	<0.001
Sleep duration ≥ 7.5 hours	0.80 (0.51, 1.08)	<0.001
Log likelihood ratio		<0.001

Age, race, educational level, smoking, drinking alcohol, diabetes, hypertension, heart disease, moderate activities, family income-to-poverty ratio, BMI, ALT, AST, HDL-C, LDL-C, triglyceride, and total cholesterol were adjusted.

## 4 Discussion

In our study sample, we discovered a nonlinear connection between the duration of sleep and the risk of depression in menopausal women. The findings demonstrated a U-shaped association between the duration of sleep and the risk of depression (sleep duration inflection point: 7.5 hours). Long duration of sleep would also increase the prevalence of depression in addition to short sleep duration.

Based on our knowledge, this is the first study to investigate the relationship between sleep duration and depression in menopausal women. Previous research indicates that estrogen deficiency during menopause may hurt sleep maintenance (26). In addition, sleep problems such as insufficient or excessive sleep may be a significant risk factor for depressive episodes in middle-aged women during menopause (27). These research findings support our conclusion that a sleep duration that is either too short or too long increases the risk of depression in menopausal women. A longitudinal study conducted in Japan revealed that insufficient sleep increases the risk of developing depression (28). Furthermore, a prospective Australian study revealed that reduced sleep duration was an independent predictor of an increase in depressive symptoms (14). Yet another prospective study (29) found no association between insufficient sleep and depression (14). The sample sizes of these studies are usually limited. In addition, the relationship between excessive sleep and depression is controversial. Most studies on long sleep duration have shown that it is unrelated to the prevalence of depression (30–32). In contrast, a recent meta-analysis suggests that long sleep duration is associated with an increased risk of depression (18). Previous meta-analyses have demonstrated that both short and long sleep durations are associated with an increased risk for depressive symptoms (33). These results broadly support the findings of this study. A recent study also found a U-shaped relationship between sleep duration and depression (34), supporting our findings. Nevertheless, some researchers only discovered a significant association between short sleep duration and depression (34, 35), most likely because they defined long sleep duration as 8 hours, which is unreasonable given that the National Sleep Foundation recommends 7-9 hours of sleep for adults (34).

Although the underlying mechanisms of the association between short sleep duration and depressive symptoms in menopausal women remain unclear, several possible explanations have been proposed. Research indicates that the prevalence of depression is higher among postmenopausal women compared to premenopausal women (4). In these women, hormonal imbalances during menopause lead to a constellation of symptoms, including insomnia, mood swings, depression, and hot flashes. There is evidence suggesting that a deficiency of estrogen during the menopausal transition may contribute to the onset of depression (4). Moreover, body fat distribution changes occur in menopausal women, with an increase in abdominal fat, which might exacerbate inflammation; proinflammatory cytokines (PICs) could potentially influence mood and mental health by disrupting the metabolism of neurotransmitters such as serotonin, dopamine, norepinephrine, and glutamate (36). Concurrently, various psychosocial factors are also implicated in the development of depression during menopause. These include relatively higher unemployment rates, retirement, and feelings of loneliness resulting from social isolation (37). Additionally, 40%-60% of menopausal women suffer from insufficient sleep due to hormonal imbalances (38). This may stem from the fluctuating estrogen levels that disrupt neurotransmitters’ metabolism, leading to sleep disturbances (39). Furthermore, abnormal levels of estrogen resulting from ovarian failure influence the sleep-wake cycle, causing a decrease in both the frequency and duration of rapid eye movement sleep, consequently contributing to reduced total sleep time (40). It has been reported that nocturnal awakenings and difficulty falling asleep are associated with lower estradiol levels (41). Other studies also demonstrate that postmenopausal women with decreased estrogen levels tend to have an increased sleep latency and reduced overall sleep time (39). Moreover, up to 80% of women experience severe hot flashes during menopause, and night sweats can lead to fragmented sleep and sleep interruptions, further curtailing sleep duration (42). These symptoms collectively highlight the intricate interplay between hormonal changes and sleep quality during the menopausal transition. Simultaneously, sleep deprivation, through its alterations to the sleep-wake cycle, can lead to disruptions in circadian rhythms, which have been linked with increased severity of depressive symptoms (43). Strong evidence suggests that short sleep duration significantly elevates levels of PICs (44), and higher PICs levels appear to increase the risk of developing depression (28). Furthermore, it is posited by scholars that sleep restriction activates the hypothalamic-pituitary-adrenal axis, thereby exacerbating depressive symptoms (45). Additionally, inadequate sleep reduces productivity, which in turn increases the likelihood of aggressive behavior and may ultimately contribute to depression and suicidal issues (46). Alternatively, it has been hypothesized that individuals with shorter sleep durations may have more time for reflective thoughts, leading to adverse outcomes associated with depression (47). Menopausal women are particularly susceptible to fatigue due to declining estrogen levels (47), and a shorter sleep period is correlated with increased daytime fatigue, which amplifies negative emotions and subsequently raises the risk of developing depression (37). In essence, the interplay between disrupted sleep patterns and hormonal changes in menopause presents a multifaceted pathway that contributes to the onset and exacerbation of depressive conditions.

At present, the mechanisms underlying the relationship between prolonged sleep duration and depression in menopausal women have not been fully elucidated. Although speculative, several potential mechanisms can be proposed to explain this association. It is well-established that menopause is a period of increased risk for depression (48). Studies show that menopausal symptoms, including hot flashes, sweating, and sleep disturbances, all influence mood changes, with depression being one of the common emotional alterations experienced during this phase (2). Moreover, in Western cultures, menopause is often perceived as a loss of sexual attractiveness, which has been linked to the development of depression (49). Furthermore, the decline in ovarian function and decreased estrogen levels during menopause are associated with a higher risk for depression (37). Moreover, in menopausal women, the decline in estrogen levels can lead to increased sleep duration (50) by activating stress response mechanisms (9). Concurrently, vasomotor symptoms resulting from decreased estrogen levels, such as hot flashes, have consistently been linked with sleep disturbances (26), which may contribute to increased sleep time. In essence, the decrease in estrogen during menopause triggers physiological and symptomatic changes that disrupt sleep patterns and potentially extend the overall sleep duration. Additionally, hormonal fluctuations during menopause can lead to disruptions in circadian rhythms (26), and longer sleep durations may be associated with such circadian rhythm disturbances (15). Research has shown that individuals with disrupted circadian rhythms are more prone to developing depression (51). Furthermore, postmenopausal women exhibit higher levels of inflammatory markers compared to premenopausal women (36), and prolonged sleep times have been correlated with elevated levels of PICs (15), which are linked to the development of depressive symptoms (52). Animal models further substantiate this connection by demonstrating that genetic disruption of circadian rhythms can induce behaviors akin to depression (51). Longer sleep durations are often associated with lower levels of physical activity, and those who sleep longer but engage less in physical activities are at a higher risk for depression (14). Moreover, extended nocturnal sleep duration is significantly related to the increased time spent in bed (33). Patients with depression might tend to spend excessive amounts of time in bed, attempting to recover from feelings of fatigue and exhaustion (33, 53); thus, complaints of oversleeping are common among depressed patients (33). Collectively, these findings provide support for our observation that prolonged sleep duration increases the risk of depression in menopausal women.

In addition, we found a significant negative correlation between sleep duration and depression scores in a population of non-smoking and diabetic menopausal women. This indicates that non-smoking and diabetic menopausal women who sleep less have higher depression scores. A meta-analysis found that smokers are 47% more likely than non-smokers to experience sleep problems (54). Inadequate sleep is strongly associated with smoking (55). In the present study, however, no correlation was found between sleep duration and depression scores among smoking menopausal women. It may be caused by chronic exposure to cigarette smoke, which interferes with the expression of estrogen in the female body, resulting in elevated estrogen levels (56). At the same time, menopausal women have an increased risk of depression due to the decline in estrogen levels, which affects sleep (9). It is possible that this interaction contributed to the lack of a correlation between sleep duration and depression in this study’s menopausal smokers. In addition, among diabetic menopausal women, the reduced hours of sleep were associated with greater levels of depression. Notably, unhealthy sleep habits are associated with an increased risk of diabetes (57). People with diabetes are more likely than non-diabetics to sleep less (58). As a consequence, diabetic menopausal women sleep too little, which increases the risk of depression.

Our research has a couple of limitations. First, this was a cross-sectional investigation; therefore, it was impossible to establish a causal relationship between depressive symptoms and sleep duration. Thus, in the future, longitudinal studies and animal experiments are needed to investigate the causal relationships in this research. Second, data on sleep duration and depression were collected through self-report, which may introduce statistical bias; however, one study found a correlation between self-reported sleep duration and objective measures (59). Thirdly, despite including many potential confounding variables in our analysis, we cannot rule out the influence of all confounding variables. Regardless of these limitations, our research has numerous benefits. Due to the use of a nationally representative sample, our research is representative of the menopausal women population in the United States. Besides this, the large number of participants that formed our study allowed us to conduct a subgroup analysis.

## 5 Conclusion

Our study found a correlation between sleep duration and depression in menopausal women. Insufficient or excessive sleep may increase the risk of depression in menopausal women.

## Data availability statement

The datasets presented in this study can be found in online repositories. The names of the repository/repositories and accession number(s) can be found in the article/supplementary material.

## Ethics statement

The studies involving human participants were reviewed and approved by the NCHS Ethics Review Board. The studies were conducted in accordance with the local legislation and institutional requirements. The participants provided their written informed consent to participate in this study.

## Author contributions

FZ: Resources, Writing – original draft. LC: Conceptualization, Writing – review & editing.

## References

[1] Stute P Spyropoulou A Karageorgiou V Cano A Bitzer J Ceausu I. Management of depressive symptoms in peri- and postmenopausal women: EMAS position statement. Maturitas (2020) 131:91–101. doi: 10.1016/j.maturitas.2019.11.002. 31740049

[2] Monfaredi Z Malakouti J Farvareshi M Mirghafourvand M. Effect of acceptance and commitment therapy on mood, sleep quality and quality of life in menopausal women: a randomized controlled trial. BMC Psychiatry (2022) 22(1):108. doi: 10.1186/s12888-022-03768-8. 35148706 PMC8840609

[3] Tang R Luo M Li J Peng Y Wang Y Liu B. Symptoms of anxiety and depression among Chinese women transitioning through menopause: findings from a prospective community-based cohort study. Fertil Steril (2019) 112(6):1160–71. doi: 10.1016/j.fertnstert.2019.08.005. 31843093

[4] Alam MM Ahmed S Dipti RK Siddiquee RE Hawlader MDH. The prevalence and associated factors of depression during pre-, peri-, and post-menopausal period among the middle-aged women of Dhaka city. Asian J Psychiatr (2020) 54:102312. doi: 10.1016/j.ajp.2020.102312. 32795954

[5] Wang XY Wang LH Di JL Zhang XS Zhao GL. Association of menopausal status and symptoms with depressive symptoms in middle-aged Chinese women. Climacteric (2022) 25(5):453–9. doi: 10.1080/13697137.2021.1998435. 34783275

[6] Kim JH Song JH Wee JH Lee JW Choi HG. Depressive symptoms, subjective cognitive decline, and subjective sleep quality are associated with slips and falls: data from the community health survey in Korean adults. Gerontology (2022) 68(5):518–28. doi: 10.1159/000518007. 35580570

[7] Xiong A Luo B Li M Chong M Wang J Liao S. Longitudinal associations between sleep quality and menopausal symptoms among community-dwelling climacteric women: A multi-centered study. Sleep Med (2022) 100:198–205. doi: 10.1016/j.sleep.2022.08.025. 36113232

[8] Otte JL Carpenter JS Roberts L Elkins GR. Self-hypnosis for sleep disturbances in menopausal women. J Womens Health (Larchmt) (2020) 29(3):461–3. doi: 10.1089/jwh.2020.8327. PMC7097677 32186967

[9] Li X Ren Z Ji T Shi H Zhao H He M. Associations of sleep quality, anxiety symptoms and social support with subjective well-being among Chinese perimenopausal women. J Affect Disord (2022) 302:66–73. doi: 10.1016/j.jad.2022.01.089. 35085670

[10] Yang Q Magnus MC Kilpi F Santorelli G Soares AG West J. Investigating causal relations between sleep duration and risks of adverse pregnancy and perinatal outcomes: linear and nonlinear Mendelian randomization analyses. BMC Med (2022) 20(1):295. doi: 10.1186/s12916-022-02494-y. 36089592 PMC9465870

[11] Creasy SA Crane TE Garcia DO Thomson CA Kohler LN Wertheim BC. Higher amounts of sedentary time are associated with short sleep duration and poor sleep quality in postmenopausal women. Sleep (2019) 42(7):zsz093. doi: 10.1093/sleep/zsz093. PMC6612671 30994175

[12] Li Y Zhao D Lv G Mao C Zhang Y Xie Z. Individual and additive-effect relationships of sleep problems and severe menopausal symptoms among women in menopausal transition. Menopause (2021) 28(5):517–28. doi: 10.1097/GME.0000000000001726. 33438893

[13] Jike M Itani O Watanabe N Buysse DJ Kaneita Y. Long sleep duration and health outcomes: A systematic review, meta-analysis and meta-regression. Sleep Med Rev (2018) 39:25–36. doi: 10.1016/j.smrv.2017.06.011. 28890167

[14] Zhai L Zhang H Zhang D. Sleep duration and depression among adults: a meta-analysis of prospective studies. Depress Anxiety (2015) 32(9):664–70. doi: 10.1002/da.2015.32.issue-9. 26047492

[15] Li J Cao D Huang Y Chen Z Wang R Dong Q. Sleep duration and health outcomes: an umbrella review. Sleep Breath (2022) 26(3):1479–501. doi: 10.1007/s11325-021-02458-1. 34435311

[16] Vanden Eng JL Chan A Abílio AP Wolkon A Ponce de Leon G Gimnig J. Bed net durability assessments: exploring a composite measure of net damage. PloS One (2015) 10(6):e0128499. doi: 10.1371/journal.pone.0128499. 26047494 PMC4457879

[17] Kim Y Son C Park YK Jo JH Park JW. Sleep duration and inflammatory mediator levels associated with long-term prognosis in temporomandibular disorders. J Oral Rehabil (2023) 50(9):830–9. doi: 10.1111/joor.13494. 37164342

[18] Sun Y Shi L Bao Y Sun Y Shi J Lu L. The bidirectional relationship between sleep duration and depression in community-dwelling middle-aged and elderly individuals: evidence from a longitudinal study. Sleep Med (2018) 52:221–9. doi: 10.1016/j.sleep.2018.03.011. 29861378

[19] Xie R Xiao M Li L Ma N Liu M Huang X. Association between SII and hepatic steatosis and liver fibrosis: A population-based study. Front Immunol (2022) 13:925690. doi: 10.3389/fimmu.2022.925690. 36189280 PMC9520084

[20] Tang Y Peng B Liu J Liu Z Xia Y Geng B. Systemic immune-inflammation index and bone mineral density in postmenopausal women: A cross-sectional study of the national health and nutrition examination survey (NHANES) 2007-2018. Front Immunol (2022) 13:975400. doi: 10.3389/fimmu.2022.975400. 36159805 PMC9493473

[21] Chaput JP Dutil C Sampasa-Kanyinga H. Sleeping hours: what is the ideal number and how does age impact this? Nat Sci Sleep (2018) 10:421–30. doi: 10.2147/NSS. PMC6267703 30568521

[22] Cai Y Chen M Zhai W Wang C. Interaction between trouble sleeping and depression on hypertension in the NHANES 2005-2018. BMC Public Health (2022) 22(1):481. doi: 10.1186/s12889-022-12942-2. 35277151 PMC8917766

[23] Kroenke K Spitzer RL Williams JB. The PHQ-9: validity of a brief depression severity measure. J Gen Intern Med (2001) 16(9):606–13. doi: 10.1046/j.1525-1497.2001.016009606.x. PMC1495268 11556941

[24] Kofod J Elfving B Nielsen EH Mors O Köhler-Forsberg O. Depression and inflammation: Correlation between changes in inflammatory markers with antidepressant response and long-term prognosis. Eur Neuropsychopharmacol (2022) 54:116–25. doi: 10.1016/j.euroneuro.2021.09.006. 34598835

[25] Chunnan L Shaomei S Wannian L. The association between sleep and depressive symptoms in US adults: data from the NHANES (2007-2014). Epidemiol Psychiatr Sci (2022) 31:e63. doi: 10.1017/S2045796022000452. 36073029 PMC9483824

[26] Lee GB Kim HC Jung SJ. Association between sleep duration and augmentation index in post-menopausal women: A moderating role of depressive symptoms. Maturitas (2021) 149:8–15. doi: 10.1016/j.maturitas.2021.04.007. 34134889

[27] Morssinkhof MWL van Wylick DW Priester-Vink S van der Werf YD den Heijer M van den Heuvel OA. Associations between sex hormones, sleep problems and depression: A systematic review. Neurosci Biobehav Rev (2020) 118:669–80. doi: 10.1016/j.neubiorev.2020.08.006. 32882313

[28] Zhong W Wang F Chi L Yang X Yang Y Wang Z. Association between sleep duration and depression among the elderly population in China. Exp Aging Res (2022) 48(4):387–99. doi: 10.1080/0361073X.2021.2008755. 34969355

[29] Yokoyama E Kaneita Y Saito Y Uchiyama M Matsuzaki Y Tamaki T. Association between depression and insomnia subtypes: a longitudinal study on the elderly in Japan. Sleep (2010) 33(12):1693–702. doi: 10.1093/sleep/33.12.1693. PMC2982740 21120150

[30] Paudel M Taylor BC Ancoli-Israel S Blackwell T Maglione JE Stone K. Sleep disturbances and risk of depression in older men. Sleep (2013) 36(7):1033–40. doi: 10.5665/sleep.2804. PMC3669078 23814340

[31] Maglione JE Ancoli-Israel S Peters KW Paudel ML Yaffe K Ensrud KE. Subjective and objective sleep disturbance and longitudinal risk of depression in a cohort of older women. Sleep (2014) 37(7):1179–187. doi: 10.5665/sleep.3834. PMC4098803 25061246

[32] Gehrman P Seelig AD Jacobson IG Boyko EJ Hooper TI Gackstetter GD. Predeployment sleep duration and insomnia symptoms as risk factors for new-onset mental health disorders following military deployment. Sleep (2013) 36(7):1009–18. doi: 10.5665/sleep.2798. PMC3669076 23814337

[33] Jiang J Li Y Mao Z Wang F Huo W Liu R. et al: Abnormal night sleep duration and poor sleep quality are independently and combinedly associated with elevated depressive symptoms in Chinese rural adults: Henan Rural Cohort. Sleep Med (2020) 70:71–8. doi: 10.1016/j.sleep.2019.10.022. 32229420

[34] Sun X Zheng B Lv J Guo Y Bian Z Yang L. et al: Sleep behavior and depression: Findings from the China Kadoorie Biobank of 0.5 million Chinese adults. J Affect Disord (2018) 229:120–4. doi: 10.1016/j.jad.2017.12.058. PMC6675597 29306691

[35] Lee MS Shin JS Lee J Lee YJ Kim MR Park KB. The association between mental health, chronic disease and sleep duration in Koreans: a cross-sectional study. BMC Public Health (2015) 15:1200. doi: 10.1186/s12889-015-2542-3. 26627637 PMC4665819

[36] Azarmanesh D Bertone-Johnson ER Pearlman J Liu Z Carbone ET. Association of the dietary inflammatory index with depressive symptoms among pre- and post-menopausal women: findings from the national health and nutrition examination survey (NHANES) 2005-2010. Nutrients (2022) 14(9):1980. doi: 10.3390/nu14091980. PMC9105364 35565951

[37] Zeng LN Yang Y Feng Y Cui X Wang R Hall BJ. The prevalence of depression in menopausal women in China: A meta-analysis of observational studies. J Affect Disord (2019) 256:337–43. doi: 10.1016/j.jad.2019.06.017. 31202988

[38] Zhou Z Yu Y Zhou R Luan R Li K. Associations between sleep duration, midday napping, depression, and falls among postmenopausal women in China: a population-based nationwide study. Menopause (2021) 28(5):554–63. doi: 10.1097/GME.0000000000001732. 33438896

[39] Verde L Barrea L Vetrani C Frias-Toral E Chapela SP Jayawardena R. Chronotype and sleep quality in obesity: how do they change after menopause? Curr Obes Rep (2022) 11(4):254–62. doi: 10.1007/s13679-022-00479-9. PMC9729134 36053414

[40] Liu X Zhang J Peng S Pei M Dai C Wang T. Mediating effects of sleep duration on the association between natural menopause and stroke risk among Chinese women. Front Neurosci (2022) 16:960497. doi: 10.3389/fnins.2022.960497. 36033607 PMC9403275

[41] Kang S Kwon DJ Hong J Go M Chung YJ Kim MR. Association of hormone therapy and changes of objective sleep quality in women of late menopausal transition with sleep disorder: a preliminary study. Menopause (2022) 29(11):1296–307. doi: 10.1097/GME.0000000000002055. 36219812

[42] Hachul H Castro LS Bezerra AG Pires GN Poyares D Andersen ML. Hot flashes, insomnia, and the reproductive stages: a cross-sectional observation of women from the EPISONO study. J Clin Sleep Med (2021) 17(11):2257–67. doi: 10.5664/jcsm.9432. PMC8636363 34170233

[43] Liu H Li D Zhao X Fang B Zhang Q Li T. Longitudinal impact of frailty states and sleep duration on subsequent depressive symptoms of older adults. J Am Geriatr Soc (2021) 69(3):1003–11. doi: 10.1111/jgs.16999 33533055

[44] Liang T Munro HM Hargreaves MK Steinwandel MD Blot WJ Buchowski MS. Patterns and correlates of sleep duration in the Southern cohort community study. Sleep Med (2020) 75:459–67. doi: 10.1016/j.sleep.2020.09.004. PMC7669688 32998092

[45] Luo Y Li Y Xie J Duan Y Gan G Zhou Y. Symptoms of depression are related to sedentary behavior and sleep duration in elderly individuals: A cross-sectional study of 49,317 older Chinese adults. J Affect Disord (2022) 308:407–12. doi: 10.1016/j.jad.2022.04.102. 35460733

[46] Choi SY Han JE Choi J Park M Sung SH Sung AD. Association between sleep duration and symptoms of depression aged between 18 and 49: the Korea national health and nutrition examination survey (KNHANES VII) from 2016 to 2018. Healthc (Basel) (2022) 10(11):2324. doi: 10.3390/healthcare10112324. PMC9690060 36421648

[47] van Mill JG Vogelzangs N van Someren EJ Hoogendijk WJ Penninx BW. Sleep duration, but not insomnia, predicts the 2-year course of depressive and anxiety disorders. J Clin Psychiatry (2014) 75(2):119–26. doi: 10.4088/JCP.12m08047. 24345733

[48] Sander B Muftah A Sykes Tottenham L Grummisch JA Gordon JL. Testosterone and depressive symptoms during the late menopause transition. Biol Sex Differ (2021) 12(1):44. doi: 10.1186/s13293-021-00388-x. 34330326 PMC8325283

[49] Hybholt M. Psychological and social health outcomes of physical activity around menopause: A scoping review of research. Maturitas (2022) 164:88–97. doi: 10.1016/j.maturitas.2022.07.014. 35964395

[50] Ormiston CK Lopez D Ishino FAM McNeel TS Williams F. Acculturation and depression are associated with short and long sleep duration among Mexican Americans in NHANES 2005-2018. Prev Med Rep (2022) 29:101918. doi: 10.1016/j.pmedr.2022.101918. 35898195 PMC9309403

[51] Zhang MM Ma Y Du LT Wang K Li Z Zhu W. Sleep disorders and non-sleep circadian disorders predict depression: A systematic review and meta-analysis of longitudinal studies. Neurosci Biobehav Rev (2022) 134:104532. doi: 10.1016/j.neubiorev.2022.104532. 35041878

[52] Zhu C Wang J Wang J Zhong Q Huang Y Chen Y. Associations between depressive symptoms and sleep duration for predicting cardiovascular disease onset: A prospective cohort study. J Affect Disord (2022) 303:1–9. doi: 10.1016/j.jad.2022.01.106. 35104466

[53] van den Berg JF Luijendijk HJ Tulen JH Hofman A Neven AK Tiemeier H. Sleep in depression and anxiety disorders: a population-based study of elderly persons. J Clin Psychiatry (2009) 70(8):1105–13. doi: 10.4088/JCP.08m04448. 19607762

[54] Otsuka Y Takeshima O Itani O Matsumoto Y Kaneita Y. Associations among alcohol drinking, smoking, and nonrestorative sleep: A population-based study in Japan. Clocks Sleep (2022) 4(4):595–606. doi: 10.3390/clockssleep4040046. 36412579 PMC9680481

[55] Chang LY Chang HY Wu WC Lin LN Wu CC Yen LL. Dual trajectories of sleep duration and cigarette smoking during adolescence: relation to subsequent internalizing problems. J Abnorm Child Psychol (2018) 46(8):1651–63. doi: 10.1007/s10802-018-0414-x. 29516340

[56] Merklinger-Gruchala A Jasienska G Thune I Kapiszewska M. Joint effect of particulate matter and cigarette smoke on women’s sex hormones. BMC Womens Health (2022) 22(1):3. doi: 10.1186/s12905-021-01586-w. 34996432 PMC8742359

[57] Wang M Zhou T Li X Ma H Liang Z Fonseca VA. Baseline vitamin D status, sleep patterns, and the risk of incident type 2 diabetes in data from the UK biobank study. Diabetes Care (2020) 43(11):2776–84. doi: 10.2337/dc20-1109. PMC7576418 32847829

[58] Han H Wang Y Li T Feng C Kaliszewski C Su Y. Sleep duration and risks of incident cardiovascular disease and mortality among people with type 2 diabetes. Diabetes Care (2023) 46(1):101–10. doi: 10.2337/dc22-1127. 36383480

[59] Monroig Ó Shu-Chien AC Kabeya N Tocher DR Castro LFC. Desaturases and elongases involved in long-chain polyunsaturated fatty acid biosynthesis in aquatic animals: From genes to functions. Prog Lipid Res (2022) 86:101157. doi: 10.1016/j.plipres.2022.101157. 35104467

